# Deciphering metabolite signalling between plant roots and soil pathogens to design resistance

**DOI:** 10.1186/s12870-025-06321-3

**Published:** 2025-03-11

**Authors:** Yee-Shan Ku, Sau-Shan Cheng, Ching-Yee Luk, Hoi-Sze Leung, Tsz-Yan Chan, Hon-Ming Lam

**Affiliations:** 1 School of Life Sciences and Centre for Soybean Research of the State Key Laboratory of Agrobiotechnology, The Chinese University of Hong Kong, Hong Kong, China; 2 Shenzhen Research Institute, the Chinese University of Hong Kong, Shenzhen, China; 3 Institute of Environment, Energy and Sustainability, The Chinese University of Hong Kong, Hong Kong, China

**Keywords:** Plant immunity, Root exudate, Microbial secretion, Plant–microbe interaction, Soil pathogen, Organic acid, Polyketide, Thaxtomin

## Abstract

Metabolites are important signaling molecules mediating plant–microbe interaction in soil. Plant root exudates are composed of primary metabolites, secondary metabolites, and macro-molecules such as organic acids. Certain organic acids in root exudates can attract pathogenic microbes in soil and promote infection. Meanwhile, secretions from soil microbes can also alter the compositions of root exudates and enhance the pathogenicity towards the target host plant. Examples of toxins in microbial secretions include polyketides and thaxtomins. The pathogenicity of plant microbes is mediated by the dynamic exchange of metabolites between the pathogen and the host plant. By deciphering this metabolite-mediated infection process, targeted strategies can be developed to promote plant resistance to soil pathogens. Examples of the strategies include the manipulation of root exudate composition and the blocking of metabolite signals that promote microbial infection. Other possibilities include minimizing the harmfulness of pathogenic microbial secretions to plants by habituating the plants to the toxin, genetically engineering plants to enhance their pathogen resistance, and treating plants with beneficial hormones and microbes. In this review, we summarized the current understanding of root exudates and soil microbe secretions that promote infection. We also discussed the strategies for promoting pathogen resistance in plants by focusing on the metabolite signaling between plants and pathogenic soil microbes.

## Background

Land plants and various microbes communicate with one another in the soil through the secretion of various molecules, including proteins, DNA, RNA, and metabolites [1–3]. For example, soil pathogens secrete effector peptides to help invade plants through their roots; microbes secrete mobile genetic elements to either inhibit or promote plant growth; legumes secrete flavonoids through their roots to attract rhizobia to establish mutualistic root nodules for nitrogen fixation [1–3]. Secretions from plant roots are termed root exudates, which are composed of various metabolites including sugars, amino acids, organic acids, and flavonoids [3]. In return, soil microbes also secrete metabolites to mediate their interactions with plants [3]. Compared to the identification of proteins and genetic elements, the identification of metabolites from plant roots and soil microbes is more technically challenging due to the diverse chemical nature of the metabolites. With the advancement of technological platforms in chromatography and mass spectrometry [4], the understanding on metabolomes is accumulating. In this review, we gathered and analyzed the latest information on metabolites in root exudates and soil microbe secretions. We also discussed the roles of metabolite signaling between plant roots and soil pathogens in the pathogenicity of soil microbes, and strategies to promote the pathogen resistance of plants based on this knowledge.

## Main text

### Root exudates contain the signaling molecules mediating plant–microbe interactions

Interactions among plants and different soil organisms are highly complex. These include mutualism between plants and beneficial microbes, competitive exclusions among soil biota, and the evolutionary arms race between plants and pests/pathogens [5]. Root exudates play a major role in mediating the ecological interactions within the soil biome by influencing the chemo-physical properties of soil and the community structure [5–7]. Root exudates include primary metabolites such as sugars and amino acids [7], secondary metabolites such as vitamins and hormones [8], and high-molecular weight compounds such as proteins [5]. Primary metabolites usually diffuse passively from root cells into the soil [7], while secondary metabolites are typically actively transported out of the root [8]. The delivery of high-molecular weight compounds may involve vesicular transport [5].

**Fig. 1:**
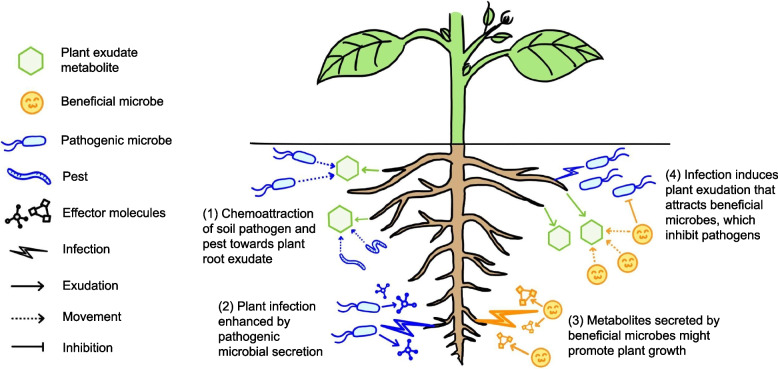
The interactions between plants and soil biota mediated by metabolite signals. Metabolites in root exudates can attract both beneficial and pathogenic microbes. Certain metabolites in root exudates can also promote pathogenic infection. Meanwhile, secretions from soil microbes can alter the composition of root exudates and enhance the pathogenicity

The composition of the root exudate is dynamic, and varies according to the plant genotype, developmental stage, and growth condition [9]. For example, upon wounding or salt treatment, the root exudates of tomato plants contained more oxylipins, a group of compounds positively correlated with the chemotrophic activity toward *Trichoderma harzianum*, which is an effective fungal strain for biocontrol [10]. When subjected to foliar infection by *Pseudomonas syringae* pv *tomato,* the model plant *Arabidopsis thaliana* exudate contained higher concentrations of long-chain organic acids and amino acids and reduced contents of short-chain organic acids and sugars. Such an alteration in the root exudate composition may create a ‘soil memory’ through its effects on the soil microbiome, and promote disease resistance in the next generation of crops [11]. The colonization by specific soil microbes can also alter the composition of root exudates. For example, the colonization by *Bacillus amyloliquefaciens* repressed the levels of lyxitol and raffinose in the root exudates of cucumber plants. Since lyxitol and raffinose favor the colonization of the pathogenic fungus *Fusarium oxysporum* f. sp. *cucumerinum*, this alteration in the root exudate composition resulted in plant growth promotion [12]. The plant–microbe interaction mediated by root exudates is illustrated in Fig. 1.

Given the agronomic significance of belowground biotic interactions, unveiling the signaling molecules that regulate these interactions will help researchers develop tools to achieve crop protection.

### Organic acids in root exudates can induce chemotaxis in soil microbes

Plant roots can exude organic acids that attract and recruit microbes to their rhizosphere. For example, tomato roots secreted malic acid, citric acid, succinic acid, and fumaric acid to induce the swarming motility and chemotactic response of the beneficial bacterium, *Bacillus amyloliquefaciens* T-5, which eventually colonized the roots of the tomato plant [13]. Malic acid and citric acid secreted by watermelon roots activated the chemotactic and swarming responses of the beneficial rhizobacterium, *Paenibacillus polymyxa* SQR-21, hence increasing its population in the rhizosphere and allowing its colonization of the watermelon root surface via biofilm formation [14]. Citric acid-containing cucumber exudates and fumaric acid-containing banana exudates were essential to induce both chemotaxis and biofilm formation in their respective beneficial rhizosphere-associated bacterial strains, *B. amyloliquefaciens* SQR9 and *Bacillus subtilis* N11 [15].

Besides beneficial microbes, organic acid exudates can also attract pathogens and induce the expression of genes related to motility, biofilm formation, and chemotaxis in these microbes. For example, cinnamic, myristic, and fumaric acids in tobacco root exudates promoted colonization and infection by *Ralstonia solanacearum* via inducing their motility-related genes *motA*, *motB*, and *filA*, and chemotaxis-related genes *CheA*, *CheW*, and *CheY* [16]. In the tomato pathogen *Ralstonia pseudosolanacearum*, a mutation in the *CheA* gene decreased its infectivity towards the tomato plant [17, 18]. The non-chemotactic *CheW* mutants of *R. solanacearum* and *CheA* mutant of *Pseudomonas fluorescens* WCS365 [18, 19] showed reduced virulence and colonization in the tomato rhizosphere. In banana, fumaric acid induced the expressions of biofilm formation-related genes *epsD* and *yqxM* in *B. amyloliquefaciens* NJN-6, and was essential for the positive chemotactic response and biofilm formation by the microbe [20]. Similar chemotaxis and enhanced virulence responses were observed in other soil-borne pests. For instance, cadaverine, putrescine, and diaminopropane in soybean and tomato root exudates served as the chemoattractant of root-knot nematodes [21]. The exogenous application of cadaverine was also shown to enhance the infection of Arabidopsis by *Meloidogyne incognita* [21].

Each chemical in the root exudate is perceived by a specific receptor in the interacting microbe. Soil microbes sense the chemical signals from plants via methyl-accepting chemotaxis proteins (MCPs) on their cell membranes. MCPs usually consist of a variated periplasmic domain for ligand accommodation and a highly conserved cytoplasmic signalling domain [22, 23]. The periplasmic domain of an MCP is flanked by two transmembrane domains for anchorage. Chemoreceptors exist in a ternary complex with the scaffold protein CheW coupling the kinase CheA to the chemoreceptors [22, 24]. In the absence of a chemoattractant, CheA undergoes autophosphorylation and in turn phosphorylates CheY. The phosphorylated CheY interacts with the flagellar rotary motor and affects the rotational direction of the motor [24–26]. As a result, the swimming and tumbling movement is altered for random directional movement [24–26]. Ligand binding at the periplasmic domain of MCPs triggers a piston-like downward displacement through the transmembrane domains to the cytoplasmic domain [24–26]. The displacement deactivates the autophosphorylation of CheA, reduces the level of the phosphorylated CheY, and renders directional swimming [24–26].

The expressions of microbial chemoreceptor genes are important for the interactions with, and the colonization of, the target plants. For example, McpA is the major chemoreceptor in *Bacillus amyloliquefaciens* SQR9 and *Ralstonia pseudosolanacearum* Ps29 for a broad range of chemoattractants, including amino acids and organic acids [17, 27]. For the nitrogen-fixing *Ensifer meliloti* (formerly named *Rhizobium meliloti* and *Sinorhizobium meliloti*), carboxylate compounds, such as acetate, propionate, and acetoacetate, are the most potent chemoattractants [28]. The carboxylates secreted by the germinating *Medicago sativa* are perceived by McpV at the cell surfaces of *E. meliloti* to mediate its chemotaxis towards the *M. sativa* rhizosphere [28]. In addition to carboxylic acids, other metabolites such as quaternary ammonium compounds (QACs), examples being betonicine, choline, glycine betaine, stachydrine and trigonelline, can also serve as chemoattractants for *E. meliloti* to migrate to the target plants upon their perception by McpX on the *E. meliloti* cell surface. Chemoreceptor mutants of *E. meliloti*, such as *mcpV* and *mcpX*, had abolished attraction towards carboxylates and QACs [28, 29]. Organic acids in root exudates are thus the potential regulatory targets for altering microbial interaction and plant–microbe interaction.

### Root exudates can promote or alleviate pathogenic infection

In addition to inducing chemoattraction, root exudates can also regulate the expression of the bacterial type III secretion system (T3SS) [30–32], which injects microbial effectors into plant cells to enhance colonization and virulence [33]. Organic acids in root exudates can either induce or repress the T3SS genes to promote or alleviate the infection [30–32]. Oleanolic acid targets T3SS through the HrpG-HrpB pathway in *R. solanacearum*, by activating the type III effector genes and accelerating disease progress in tobacco [31]. Other organic acids, such as *o*-coumaric acid (OCA) and *t*-cinnamic acid (TCA), that are enriched in tobacco root exudates were also found to induce the expressions of *dspE*, *hrpA* and *hrpN*, which encode the T3SS effector, T3SS pilus, and T3SS harpin respectively [32]. On the other hand, benzoic acid exuded by tobacco roots [34] suppressed T3SS in the bacterium *Erwinia amylovora* that causes fire blight, thereby alleviating the hypersensitive response in tobacco [30].

The antimicrobial activities of organic acids via other biochemical mechanisms have also been reported. Benzoxazinoid derivatives, which are a group of cyclic hydroxamic acids, are commonly found in the root exudates of plants belonging to the family *Poaceae*. For example, 2,4-dihydroxy-7-methoxy-1,4-benzoxazine-3-one (DIMBOA) was found in the root exudates of *Secale cereale* L., *Triticum aestivum* L. and *Zea mays* L. [35]. DIMBOA and its derivatives have antimicrobial activities against *R. solanacearum*, which causes bacterial wilt [36]. At the same time, DIMBOA could also recruit plant-beneficial microbes, such as *Pseudomonas putida* KT2440, showing affinity towards the DIMBOA-containing maize root exudate, along with the increased transcriptional activity of benzoate catabolism- and chemotaxis-related genes [35].

The versatile effects of root exudates on different microbes are dependent on the different signaling cascades in the microbes. Therefore, detailed studies on the signalling events underlying the root-exudate mediated plant–microbe interaction will be essential for pathogen control.

### Soil pathogens induce the secretion of root exudates

Sensing and reacting to the chemical signals emitted by soil pathogens is integral to plant defense. Various microbial secretions have been shown to elicit alterations in the root exudate composition, and the exudation can be an adaptive immune response [37]. In sweet basil, upon the challenge by the pathogenic fungus *Pythium ultimum* or the pathogenic bacterium *Agrobacterium rhizogenes,* rosmarinic acid (RA) was induced and released from the hairy roots [37]. RA, absent in the root exudate of uninfected plants, was shown to disrupt, intercept, and convolute the cell surface of another fungal pathogen *Aspergillus niger*. RA was also shown to disrupt biofilm formation by inhibiting quorum sensing [38]. However, concentration matters. Upon *Pseudomonas aeruginosa* infection, although sweet basil roots secrete RA, the concentration is not enough to inhibit biofilm formation by the microbes [39]. Thus, the infection is mortal despite the secretion of RA [39]. In tobacco plants, the bacterial wilt-causing pathogen, *R. solanacearum*, induced the root exudation of caffeic acid, which has been shown to disrupt the cell membrane of *R. solanacearum* [40]. Moreover, caffeic acid could repress the expression of biofilm-forming genes, *lecM* and *espsE*, while activating *phenylalanine ammonia-lyase* (*PAL*) and *peroxidase* (*POD*) to promote the accumulation of lignin and hydroxyproline for greater plant resistance against bacterial infection [40]. Upon infection by the pathogenic oomycete *Pythium ultimum*, barley roots secreted phenolic and organic acids, including canillic, p-coumaric, and fumaric acids [41]. Similarly, when challenged by *Fusarium graminearum*, the levels of antifungal phenolics such as t-cinnamic, p-coumaric, ferulic, syringic, and vanillic acids were increased in barley root exudates [42]**. **The induction of root exudates by the surrounding microbes adds one more layer to the root-exudate mediated plant–microbe interaction. When manipulating root exudates, such a dynamic process has to be taken into account.

### Microbial secretions can enforce the pathogenicity

As early as 1935, the stimulation of sporangium production in *Phytophtora* sp. with a non-sterile soil extract was demonstrated to show the pathogenicity of microbial secretions [43]. Since then, the metabolic diffusates of soil micro-organisms have been found to promote sporangium formation in *Phytophthora cinnamomi*, which is a plant pathogen capable of infecting close to 5,000 plant species [44–46]. Efforts have been applied to discover the pathogenic components in the cell-free extracts of microbial cultures. For example, an autoclaved soil water extract was shown to promote the colonization of rhododendron leaf discs by *Phytophthora nicotianae*. The phenomenon suggests that the virulence effector is heat-stable [47]. The five major bacterial components of the soil water extract were then isolated. Among them, *Bacillus megaterium* Sb5 was found to have a similar infection-promoting effect to the soil water extract on the colonization of rhododendron leaf discs by *Phytophthora nicotianae* [47]. Furthermore, the cell-free filtrate (CFF) of *Bacillus megaterium* Sb5 was shown to be responsible for this effect, the majority of which could be attributed to the > 3-kDa fraction of the CFF [47]. However, the exact identity of the effector molecule has remained unknown [47]. These early observations bring forth the subsequent investigations on infection-promoting metabolites from microbes. The identification of the infection-promoting metabolites allows specific control of the microbial pathogenicity.

### The infection-promoting metabolites in microbial secretions

The proteins and nucleic acids secreted by pathogenic soil microbes to promote the infection of plants have been previously summarized [1, 2]. In addition to these molecules, soil microbes also secrete various metabolites to promote the infection of their target plants. In this review, we focus on polyketides and thaxtomin which are common metabolites secreted by root pathogens of plants (Table 1).

**Table 1:** Examples of infection-promoting metabolites in microbial secretions

Metabolites	Examples of microbial sources and functions	References
Polyketides	Fungal pathogen *Bipolaris maydis* (also known as *Cochliobolus heterostrophus*) • T-toxin C_41_ polyketides underlie the virulence to T-maize • Synthesis controlled by genes on Tox1A locus *(PKS1*, *PKS2*, *LAM1*, and *OXI1*) and Tox1B locus (*DEC1*, *RED1*, *TED2*, and *RED3*)	[48, 49]
	Fungal pathogen *Fursarium oxysporum* • Virulent isolates secrete nonaketides, including naphthazarin quinones, bikaverin and norbikaverin, as well as haptaketides, including naphthoquinones, nectriafurone, andydrofusarubin lactol, and 5-*O*-methyljavanicin	[50]
	Fungal pathogen *Verticillium dahliae* • Contains polyketide synthetic genes which were horizontally transferred from *F.oxysporum*	[51]
	Fungal pathogen *Fusarium virguliforme* • The expressions of polyketide synthetic genes including those encoding snoal-like polyketide cyclase protein and lovastatin-like diketide synthase were found to be upregulated upon the infection of soybean root	[52]
Thaxtomin	Bacterial pathogen *Streptomyces scabies* • Thaxtomin A & B were isolated from infected potato tuber, though the mode of infection was unknown • Thaxtomin A production and virulence controlled by several *bld* genes (global regulators of Streptomyces growth morphologies) and thaxtomin biosynthetic genes (*txt*)	[53, 54]

#### Polyketides

Polyketides are molecules with a carbon skeleton, and they are secondary metabolites found in bacteria, fungi, and plants. Examples include polyphenols, polyenes, polyethers, enediynes, and macrolides [55]. Genes encoding polyketide synthases have also been identified in microbes including plant pathogens [56–58]. Polyketides are widely used in both drug and agricultural industries for their antibacterial and antifungal activities [48, 49]. However, polyketides from plant pathogens can cause chlorosis and inhibit growth of the host plants [50]. *Pseudomonas* is a common plant pathogen, it secretes the polyketide coronatine, which structurally mimics the plant hormone jasmonate (JA) and represses JA-mediated defense responses [51].

In early days, before being known as polyketides, T-toxin was isolated and identified as the toxin produced by race T of the foliar pathogen *Bipolaris maydis* (formerly known as *Helminthosporium maydis*), the causal agent of the epidemic corn blight in 1970–1971 [52]. Later, T-toxin was characterized as a family of molecules mainly consisting of C_41_ polyketides [52, 53, 59], and the cause of the virulence of race T toward male-sterile (T) maize [53]. *PKS1*, encoding a polyketide synthase, is associated with the production of T-toxin and the virulence of race T [53]. It was found that at least nine genes, including *PKS1* (*polyketide synthase 1*), *PKS2* (*polyketide synthase 2*), *LAM1* (encoding a 3-hydroxyacyl-CoA dehydrogenase-like protein) and *OXI1* (encoding a dehydrogenase) mapped to the *Tox1A* locus, *DEC1* (encoding a decarboxylase), *RED1* (encoding a ketoreductase), *RED2* (encoding a ketoreductase) and *RED3* (encoding a ketoreductase) mapped to the *Tox1B* locus, and an additional gene encoding an unknown protein, were involved in the biosynthesis of T-toxin [53, 54].

Root pathogens also secrete polyketides to invade plants. *Fursarium oxysporum* is a prevalent fungal pathogen found on the root surface of cotton. It causes wilting and yield loss of cotton. Most *F. oxysporum* f sp *vasinfectum* isolates which are virulent to Upland cotton (*Gossypium hirsutum* L.) secrete nonaketides, including naphthazarin quinones, bikaverin and norbikaverin, as well as haptaketides, including naphthoquinones, nectriafurone, andydrofusarubin lactol, and 5-*O*-methyljavanicin [60]. However, *F. oxysporum* f sp *vasinfectum* isolates which could not cause disease of Upland cotton (*G. hirsutum* L.) failed to synthesize or secrete the haptaketides [60]. Polyketides are therefore suggested to be the targets for cotton disease control. In a genomic study on *Verticillium dahliae*, which invade plant roots, polyketide synthase gene homologs were found to be horizontally transferred from *F. oxysporum* f sp *vasinfectum* to *V. dahlia* [61]. In a separate study on the soybean pathogen *Fusarium virguliforme* which causes root necrosis, polyketide synthetic genes including those encoding snoal-like polyketide cyclase protein and lovastatin-like diketide synthase were found to be upregulated in the pathogen [62]. These studies showed the importance of polyketides to plant infection and demonstrated the use of sequencing platforms to study the polyketide-related virulence.

#### Thaxtomin

Thaxtomin is a well-known phytotoxin in *Streptomyces*, which is a large genus of plant pathogens that infect potato and other taproot plants [63]. Thaxtomins were first identified as secondary metabolites in *Streptomyces scabies* infected potato slices [64]. They were then known for their inhibitory effects on cellulose synthesis, giving similar symptoms to plants treated with known cellulose biosynthesis inhibitors including dichlobenil and isoxaben [65]. Specifically, thaxtomin A was shown to inhibit the incorporation of glucose for cellulosic cell wall formation [66].

In an attempt to isolate the phytotoxins associated with the pathogenicity of *S. scabies* to potato, Thaxtomin A and Thaxtomin B, characterized as 4-nitroindol-3-yl-containing 2,5-dioxopiperazines, were isolated from *S. scabies*-infected potato tubers [64, 67]. Since Thaxtomin A is produced in an amount 20 folds greater than Thaxtomin B, it is regarded as the major phytotoxin associated with *S. scabies* [68]. The production and virulence of Thaxtomin A are regulated by several *bld* genes, which are highly conserved among *Streptomyces* spp. and are crucial for the regulation of normal morphological development of the fungi [69, 70]. The *S. scabies* mutants *ΔtxtA*, *ΔbldA*, *ΔbldC*, *ΔbldD*, *ΔbldG*, and *ΔbldH* had significantly reduced production of Thaxtomin A compared to the wild type, *S. scabies* 87–22 [69]. Compared to those treated with the wild type, radish seedlings treated with *ΔtxtA*, *ΔbldA*, *ΔbldC*, *ΔbldD*, *ΔbldG*, or *ΔbldH* all had longer root and shoot lengths, which suggested the better growth and reduced toxicity [69]. The *S. scabies* mutants also led to reduced virulence phenotypes on potato tuber tissue compared to the wild type [69]. Gene expression analyses showed that the *ΔbldA*, *ΔbldC*, *ΔbldD*, *ΔbldG*, and *ΔbldH* mutants all had reduced expression levels of *txtR*, *txtA*, and *txtD* [69]. *txtR* encodes a cluster-situated regulator, TxtR, which activates the expressions of thaxtomin (txt) biosynthetic genes in response to cello-oligosaccharides [69]. *txtA* encodes the nonribosomal peptide synthetase responsible for producing the thaxtomin backbone while *txtD* encodes a nitric oxide synthase, which is needed for the nitration of thaxtomin A [69]. Understanding the mechanisms of toxin secretion will facilitate the development of strategies to control the pathogenicity.

The interactions between plants and soil biota mediated by metabolite signals are summarized in Fig. 1.

### Promoting plant resistance against soil pathogens by manipulating the root exudate

As discussed  above,  Main text metabolites in the root exudate could mediate plant–microbe interactions, induce chemotaxis, and promote pathogenic infection. Therefore, the manipulation of root exudates could be an effective strategy to enhance the plant resistance to soil pathogens, either by changing the exudate composition or by blocking the metabolite signal from the root exudate, or both (Table 2).

**Table 2:** Methods to promote pathogen resistance through the manipulation of root exudates

Methods of root exudate manipulation	Examples	Things to consider in field applications	References
Manipulating root exudate composition	Controlling the release of root exudates to soil by altering the expression patterns of transporters using mutant plants (i.e., citrate-, malate- and glutamate-derived γ-aminobutyric acid [GABA] released from the respective MATE and ALMT activities)	• Target site of exudation on root tissue • Carbon pool and resident rhizosphere community in soil	[71]
	Altering the phenolic profiles of the *Abelmoschus esculentus* root exudate by priming the plant with the endophyte *Alcaligenes faecalis* strains BHU 12, BHU 16, or BHU 17. The colonization by the endophytic strains protected the host plant against pathogen attack Examples of root exudate components enhanced by the endophyte treatments: shikimic acid, gallic acid		[72]
	Manipulating the biosynthesis of special root exudates in *Arabidopsis thaliana* by reconstituting three evolutionarily divergent triterpene biosynthetic pathways, which could selectively modulate the growth of Arabidopsis-specific microbiota Examples of genes for manipulation: *THAR1* and *THAR2* encoding oxido-reductases, *THAA2* encoding thalianol acyltransferase 2		[73]
Blocking metabolite signals	Applying biochar with or without tomato root exudates to adsorb pathogen directly or indirectly, to lower the pathogenic microbe swarming motility and to reduce pathogen colonization on the root, eventually suppressing the bacterial wilt disease	• Particle size and pore size of biochar (related to the size of the pathogen concerned, and the specific surface area for pathogen adsorption) • Biochar production condition (related to the stability of the physical and chemical properties of biochar) • Type of crop • Soil conditions • The undesired effect to block beneficial microbes	[74]
	Applying the wrap-and-plant technology to potato using the lignocellulosic matrix (LCM), which strongly adsorbs the root exudate components to the cellulose, thereby disrupting the host-locating, egg-hatching and maturation processes of potato cyst nematodes (PCNs), eventually improving the potato yield		[75]

#### Changing the root exudate profile

For years, efforts have been made to eliminate soil-borne pathogens in crop disease management, but the outcomes were not always satisfactory. Soil pathogens in the field are difficult to control as they can reside in the soil as saprophytes for a prolonged period until a favorable environmental condition arises [76]. Besides, the traditional method of applying pesticides, nematicides or bactericides has poor penetration into the soil matrix and therefore is usually insufficient to completely inhibit the pathogens or pests [73]. In addition, a generous application of these biocides may lead to environmental pollution or cause public health concerns [77].

New approaches of constructing a rhizobiome profile that facilitates disease resistance in plants have been proposed, for example, by inoculating the plant with a synthetic microbial community as the biocontrol for specific pathogens [78]. Multiple subsequent studies have suggested that complex inocula with species-rich communities provide plants with much enhanced disease resistance compared to single-strain inocula [71, 78, 79]. However, the approach presents a tremendously greater difficulty in designing the appropriate synthetic microbial community to achieve a desirable and controllable outcome for the host plant.

Another recent approach to enhancing disease resistance in plants is the transplantation of the soil and microbes associated with the rhizosphere of a resistant plant to the susceptible variety, or those from the rhizosphere of a healthy plant to the next generation [80, 81]. However, microbial inocula are often inconsistent and short-lived in the field due to unfavorable soil conditions and competition with existing microbes in the soil [72]. Therefore, instead of exploiting the microbiome as inoculants to promote soil pathogen resistance, a more efficient alternative solution, perhaps in the form of manipulating the root exudate profile, is needed.

The ability of root exudates to shape the root microbiome has been proven in previous studies [74, 75, 82, 83]. The alteration of root exudate compositions by modulating the gene expression patterns of specific metabolic pathways or transporter proteins may then provide a more consistent selection pressure on the rhizobiome [84]. In order to engineer a root exudate profile that can specifically suppress a pathogen in a certain host plant, knowledge of the respective roles of different root exudate components in affecting microbial activities is important. To date, studies using mutant plants with distinctive variations in the exudate profile have helped to identify and characterize the roles of different exudate components in the plant–microbe relationship. For example, the mutation of *ABCG30*, which encodes an ATP-binding cassette (ABC) transporter in Arabidopsis, led to the altered root exudate profile with increased phenolics but decreased sugars [85]. Such an alternative is associated with the change of fungal and bacterial communities in the surrounding soil [85]. In another study, the mutations of triterpene synthetic genes in Arabidopsis were also shown to alter the composition and diversity of root microbiota [82]. In addition to phenolics, sugars, and terpenes, the levels of root-exudated molecules such as coumarins and benzoxazinoids are also suggested to be associated with the rhizospheric microbiota [86]. The secretions of roots to shape the rhizospheric microbia are illustrated in Fig. 1. More examples are extensively reviewed and summarized in another review [86].

Experiments on modulating root exudate profiles to recruit beneficial or host-specific microbes have also been performed [74, 82, 87]. It was found that phenolics in the root exudate of *Abelmoschus esculentus* attract endophytic *Alcaligenes faecalis* for the activation of defense responses against pathogenic microbes [87]. It was discovered that individual phenolic acids at different concentrations could differentially affect the potential of bacterial strains in chemotactic movement and biofilm formation. A further alteration in the phenolic profile of plant exudates after the colonization by *A. faecalis* helped in recruiting even more beneficial microbes to the rhizosphere and fortified the plant against disastrous soil-borne pathogens such as *Sclerotium rolfsii* [87]. Gene mutation can lead to altered root exudation profiles, which affect the rhizospheric microbiota composition. In other words, different genotypes could be associated with altered pathogen resistance ability of plants in soil. Indeed, in wheat, genotypes corresponding to the key steps in domestication were shown to have different root exudate profiles [88]. The search for natural crop varieties or the generation of new varieties carrying desirable genes by breeding could be the strategies to promote pathogen resistance of crops via the alternation of root exudate profiles.

To answer the question of how plants modulate and assemble microbiota specific to their rhizobiomes, three divergent pathways in *Arabidopsis thaliana* for root triterpene metabolite biosynthesis were constructed and evaluated with respect to the ability of these specialized metabolites to recruit host-specific microbiota [82]. The three divergent tripterpene pathways are responsible for the syntheses of thalianin, thalianyl fatty acid ester, and arabidin respectively [82]. Genes including *THAR1* and *THAR2* encoding oxido-reductases, as well as *THAA2* encoding thalianol acyltransferase 2 are important for the synthesis of the triterpenes [82]. The specialized triterpene compounds could selectively modulate the growth of *A. thaliana* root microbes, shaping a rhizobiome profile distinctive from taxonomically remote species such as wheat and rice. This study suggested that plant could assemble a species-specific microbial community for their own benefits with specialized metabolic pathways [82]. The selection of distinct bacterial populations with engineered root exudate compositions was also demonstrated using transgenic *Lotus* plants which produce opines [74].

However, one study discovered that soil type, root type (nodal or seminal) and position (base or apex) may have greater influences on the root microbiome than does the modulation of exudate profiles [84]. The gene expression patterns of transporters from the aluminum-activated malate transporter (ALMT) and the multidrug and toxic compound extrusion (MATE) families were altered in rice and wheat in order to change their root exudate compositions, and the subsequent shifts in the microbiome profiles along the root system in different soil types were examined [84]. The spatial variations in the rhizobiome added to the complexity in constructing an artificial root exudate profile, where a specific region in the root system may have to be targeted for exudate profile manipulation in order to achieve the disease resistance desired.

In the natural environment, root exudates mediate soil nutrient solubilization and toxic compound chelation [89]. Root exudates also impact on plant diversity in the habitat and soil functioning such as nutrient decomposition and recycling [90]. The alteration of root exudate profiles may thus threaten the soil ecosystem. Therefore, approaches that specifically address targeted compounds in root exudates will be needed to minimize the threats, and the understanding on plant–microbe interactions through these compounds will facilitate the development of suitable measures.

The methods used in promoting the resistance of plants to soil pathogens by root exudate manipulation are summarized in Table 2.

#### Blocking the metabolite signal

Besides altering the root exudate profile, another way to combat infection is to block the signal transduction between plant roots and pathogens. Apart from being energy-rich resources, root exudates can also enhance pathogen virulence by triggering the germination and sporulation of pathogens, and acting as a signal for chemotaxis [91–94].

The use of biochar could be a potential tool to disrupt the plant-pathogen interaction. Biochar is produced by the thermal degradation of organic matter such as wood biomass and agricultural crop residues under low-oxygen conditions [95]. The porous structure and the large surface area of biochar makes it an effective adsorbent of root exudates [96]. Researchers have found that the application of biochar on tomato can significantly alleviate bacterial wilt caused by *Ralstonia solanacearum*, a bacterial pathogen that can infect a broad range of host species, including economically important crops such as potato and tomato [91, 97]. Biochar might have been able to reduce infection by pathogens indirectly through the adsorption of root exudates that promote pathogen chemotaxis. The removal of tomato root exudates from the soil through biochar adsorption further reduced pathogen infection by intensifying the resource competition within the bacterial community in the soil [91]. Even in the absence of root exudates, biochar was shown to be able to directly adsorb pathogens and suppress their swarming motility, rendering it a cheap and environmentally friendly tool for disease control in the field [91]. Interestingly, biochar may have the potential to also disrupt microbial communications. A study has found that the autoinducer sorption property of biochar greatly reduced the availability of acyl-homoserine lactone (AHL) for cell-to-cell communications [98]. AHL is a family of signaling molecules involved in quorum sensing and intraspecies communication in many gram-negative bacteria, including the pathogens responsible for soft rot in plants and nitrogen-fixing bacteria [99, 100].

Despite the potential benefits of biochar in disease control, we should be cautious of its application in the field. The type of crop and the soil condition should be considered. For example, when handling legume plants, root exudates are needed to attract beneficial microbes such as Rhizobia. Moreover, evidence has shown the possibility of biochar to turn mutualistic mycorrhizal fungi into parasitic ones under high soil nitrogen levels [101].

Another promising tool using the same principle of root exudate adsorption with proven success in the field is the lignocellulosic matrix (LCM) of banana fiber. The wrap-and-plant technology (W&P) using LCM has significantly increased the potato yield in Kenya by controlling potato cyst nematodes (PCNs) caused by *Globodera rostochiensis* [93]. Through the adsorption of potato root exudates in the banana-fiber LCM, the chemo-attraction of the PCN to the plant was disrupted, resulting in reduced hatching, impeded chemotaxis and delayed maturity of the PCN [93].

Using the same principle of exudate adsorption, both biochar and LCM could impede the invasion of plants by pathogens, through different methods of application and material design. Biochar that is spread on the soil to adsorb root exudates directs pathogens away from the plant root, while the wrapping of LCM around seed potatoes strongly adsorbs and retains exudate compounds, such as the hatching factor α-chaconine, via hydrogen bonds and inter-molecular bonds in the matrix, thus rendering them unable to be sensed by the PCN [91, 93]. Both approaches disrupt plant–microbe interaction by absorbing microbial secretions and root exudates. The blocking of plant-growth promoting microbial interactions is foreseen. Therefore, measures that specifically address target compounds will be needed to minimize the unwanted side effects.

### Promoting pathogen resistance by minimizing the harm from pathogenic microbial secretions

While the strategy of altering or intercepting the metabolite signals from plants seems promising for promoting pathogen resistance, manipulating the metabolite signals from soil microbes is more challenging due to the diversity of the microbiome in soil. Nevertheless, understanding the microbial metabolite signals can provide directions on how to interrupt the signal perception by plants (Table 3).

**Table 3:** Methods of promoting pathogen resistance by minimizing the harm from pathogenic microbial secretions

Harm reduction approaches	Examples	References
Habituation of plants to toxic microbial metabolites	Treatment with a gradually increasing level of thaxtomin A (from 0.1μM to 1.3 μM over a period of 12 months) on a polar cell suspension culture promoted the resistance to thaxtomin A (2 μM), dichlobenil (5 μM), and isoxaben (5 μM) The habituated cells had altered expressions of genes related to cell wall synthesis, lignin and flavonoid synthesis, and DNA and chromatin modifications • Examples of genes having downregulated expressions: • Cell wall related: genes encoding glycosyl hydrolase, xyloglucan endotransglucosylases/hydrolases, polygalacturonases • Phenylpropanoid pathway: genes encoding cinnamyl-alcohol dehydrogenase, caffeic acid/5-hydroxyferulic acid O-methyltransferase, trans-caffeoyl-CoA 3-O-methyltransferase, hydroxycinnamoyl-Coenzyme A shikimate/quinate hydroxycinnamoyltransferases • Examples of genes having upregulated expressions: • Cell wall related: genes encoding beta-xylosidase, beta-mannan endophydrolase, polygalacturonase, pectinesterase • DNA and chromatin modifications related: genes encoding histone H1, H2, H3, H4	[88]
	Potato tubers regenerated from calli habituated with thaxtomin A had enhanced resistance to thaxtomin A and *Streptomyces scabiei*	[89]
Genetic engineering of plants	Under thaxtomin treatment, Arabidopsis *txr1* mutant seedlings were longer than the wild type	[90]
Treating plants with beneficial hormones or microbes	The auxin 2,4-dichlorophenoxyacetic acid (2,4-D) spray on potato Russet Burbank tuber promoted the resistance to *Streptomyces scabiei* and thaxtomin A Treatment details: foliar spray 14 days after tuber initiation; both single spray at 0.9 mM and three sequential sprays at 0.9 mM, 10-day intervals, were effective to promote resistance	[93]
	2,4-D and IAA promoted the resistance of Arabidopsis to thaxtomin A Treatment details: 2,4-D: surface-sterilized seeds were onto the surface of plates of MS (Murashige and Skoog) medium added with 0.1 μM thaxtomin A, and with or without 2,4-D (0.1, 0.2, or 1.0 μM) treatments; 25 day growth interval IAA: Seedlings were grown for five days on MS medium before excision of stem segments (lacking apical meristems to remove major sources of endogenously produced IAA). The seedlings were then plated onto MS medium amended with thaxtomin A (0.1 μM) with or without IAA (0.01, 0.1, or 1.0 μM) treatments. The level of chlorosis was observed five days after the treatments	[93]
	The inoculation of Arabidopsis with thaxtomin A-deficient endophytic *Streptomyces* sp. IFB-A02 and IFB-A03 enhanced the plant’s resistance to *Streptomyces scabies* Treatment details: Six-week-old plants were pre-inoculated with IFB-A02 or IFB-A03 before being attacked by *Streptomyces scabies*. The disease symptom was observed five days after the attack	[94]

#### Habituation of plants with toxic microbial metabolites

Thaxtomin A is the major toxin secreted by *Streptomyces scabies* to cause potato common scab. The promotion of resistance to thaxtomin A by habituation was first demonstrated in a poplar cell suspension culture [102]. In the study, the cell culture was treated with a gradually increasing level of thaxtomin A over a period of 12 months. Compared to the control cells without thaxtomin A treatment, the treated cells had reduced size and slower growth. However, the treated cells were more resistant to thaxtomin A, which inhibits cellulose synthesis. The habituated cells had an altered cell wall composition with a reduced level of cellulose but an increased level of pectin. Using GeneChip, the habituated cells were shown to have altered expressions of genes related to cell wall synthesis modification, lignin and flavonoid synthesis, and DNA and chromatin modifications. In addition to thaxtomin A, the habituated cells were also more resistant to dichlobenil and isoxaben, which are also inhibitors of cellulose synthesis. The regeneration of potato plants from thaxtomin A-habituated calli was later reported [103]. In the study, potato Russet Burbank calli were habituated with thaxtomin A and regenerated into plants that produced tubers [103]. In a growth chamber, these regenerated tubers were infected with *S. scabiei*. Compared to those tubers from the untreated parent cultivar, the regenerated tubers from the thaxtomin A-habituated calli were more resistant than those from the parent cultivar to the infection, as well as to thaxtomin A. In the field, the tubers of the plants regenerated from thaxtomin A-habituated calli were also more resistant to scab infection compared to the parent cultivar [103].

#### Genetic engineering of plants

The model plant Arabidopsis is typically sensitive to thaxtomin. In a screen of Arabidopsis mutants, a mutant having increased resistance to thaxtomin was identified. By map-based cloning, the mutated gene was found to be *TXR1*, which is related to the transport of thaxtomin into plant cells [66]. Compared to the wild type, the *txr1* mutant had reduced uptake of thaxtomin upon treatment with the chemical. Although *txr1* mutant seedlings were shorter than the wild type without thaxtomin treatment, they were taller than the wild type when under thaxtomin treatment [66].

#### Treatment with a beneficial hormones or microbes

Since tissue culture and genetic engineering are more technically demanding, treatment with a growth-promoting hormone or microbe appears to be an easier approach to promoting pathogen resistance. An auxin 2,4-dichlorophenoxyacetic acid (2,4-D) spray on potato Russet Burbank tubers promoted the resistance to *S. scabiei* as well as to thaxtomin A in the potato tubers [104]. Using Arabidopsis as another model, 2,4-D and indole-3-acetic acid (IAA) applications were also shown to promote the resistance to thaxtomin A [104]. Although the detailed mechanism of how the auxins promoted the resistance was unclear, the study suggested that auxins might promote the resistance by alleviating the toxicity of thaxtomin A.

The application of beneficial microbes has been suggested as a greener alternative to chemical fertilizers. The inoculation of Arabidopsis with thaxtomin A-deficient endophytic *Streptomyces* sp. enhanced the plant’s resistance to the pathogenic *Streptomyces scabies* [105]. IFB-A02 and IFB-A03 are endophytic *Streptomyces* strains that could colonize the root and stem of Arabidopsis [105]. The pre-inoculation of IFB-A02 or IFB-A03 into Arabidopsis promoted the accumulation of ROS upon infection by *S. scabies*. ROS are signaling molecules that elicit disease resistance responses in plants upon pathogen infection [105]. IFB-A03 pre-inoculation also induced the expressions of systemic acquired resistance (SAR) marker genes, *PR-1* and *PR-5*, in the Arabidopsis plants [105]. The phenomenon is similar to salicylic acid (SA) treatment, which also induced the expressions of the SAR genes. In the SA-deficient Arabidopsis mutant *eds5*, the expression of the defense gene *PDF1.2* was repressed compared to the wild type. However, IFB-A03 treatment rescued the *PDF1.2*-suppressed phenotype in the *eds5* mutant. The study suggested that IFB-A03 promoted the resistance to *S. scabies* through an SA-dependent pathway [105].

**Fig. 2:**
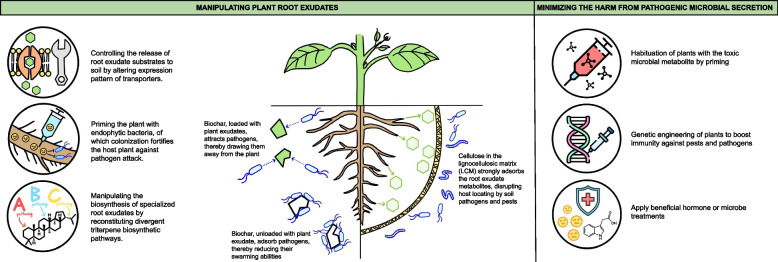
The methods to promote pathogen resistance of plants by manipulating the metabolite signals. The promotion of the resistance can be achieved by manipulating plant root exudates or minimizing the harm from pathogenic microbial secretion. The strategies to manipulate plant root exudates include the control of root exudate release and the production of specialized root exudates by genetic engineering. The resistance can also be achieved by blocking the metabolite in root exudate that attracts pathogenic microbes. The strategies to promote the resistance also include the minimization of pathogenicity of microbial secretions, which can be archived by priming the plants with the toxic microbial metabolite, genetic engineering the plants, and the application of beneficial hormone or microbes

The use of beneficial hormones or microbes offers a green and easy alternative for disease control. However, more research on the pathogenicity mechanism will be needed to ensure the specificity and avoid unpredicted side effects on plant growth and the ecosystem. The methods to promote pathogen resistance of plants by manipulating the metabolite signals are summarized in Fig. 2.

## Conclusions

Root-microbe interactions in soil are dynamic processes mediated by both root exudates and microbial secretions. Root exudates can attract soil microbes while microbial secretions can promote the infection of the target plant. Microbial secretions can also alter the compositions of root exudates. Metabolites are a major component of both root exudates and microbial secretions. Plant-driven disease control strategies targeting plant-pathogen communications can focus either on manipulating the root exudate profile or on disrupting the signal transduction between the plant host and the pathogen in the soil. The design of practical applications to enhance plants’ resistance to soil pathogens in the field requires multiple levels of consideration, including the physical and chemical properties of the soil (pH and nutrient levels), the resident microbiome (competitions among microbes and the balance within communities), the temporal (growth stage) and spatial (root tissue and region) parameters of the plant, and the type of crop (legume or tuber). Disease control can also be achieved by minimizing the harm from the pathogenic microbial secretions, through habituating plants to toxic microbial metabolites, genetically engineering plants to reduce their uptake of the toxins, and treating plants with growth-promoting hormones or microbes to boost their pathogen resistance. Since the toxicity of microbial secretions is usually dose-dependent, case-by-case strategies will be required to promote plant resistance against soil pathogens. Previous studies have revealed the correlation between genotypes and rhizospheric microbiota. Based on the knowledge, it is also possible to employ crops with desirable genotypes to establish pathogen resistant varieties by approaches including the selection of natural mutants, breeding, and genetic engineering. By understanding the mechanisms behind the pathogenicity, we will be better able to devise specific measures to improve plant resistance to specific pathogens.

## Data Availability

Not applicable.

## Abbreviations

T3SS: Type III secretion system
OCA: *O*-Coumaric acid
TCA: *T*-Cinnamic acid
DIMBOA: 2,4-Dihydroxy-7-methoxy-1,4-benzoxazine-3-one
Txt: Thaxtomin
ROS: Reactive oxygen species
PCD: Programmed cell death
ALMT: Aluminum-activated malate transporter
MATE: Multidrug and toxic compound extrusion
AHL: Acyl-homoserine lactone
LCM: Lignocellulosic matrix
W&P: Wrap-and-plant technology
PCN: Potato cyst nematode
GABA: Glutamate-derived γ-aminobutyric acid
2,4-D: 2,4-Dichlorophenoxyacetic acid
IAA: Indole-3-acetic acid
SAR: Systemic acquired resistance
